# Supplementary material for: Control of the Organization of 4,4′-bis(carbazole)-1,1′-biphenyl (CBP) Molecular Materials through Siloxane Functionalization
Source: Molecules. 2023 Feb 21;28(5):2038. doi: 10.3390/molecules28052038 (PMC10003964; doi:10.3390/molecules28052038)
Supplement: Supplementary file 1 [file molecules-28-02038-s001.zip › molecules-2207299-supplementary.pdf]

Suppporting Information

Control of the organization of 4,4′-bis(carbazole)-1,1′-biphenyl (CBP) molecular materials through siloxane functionalization

Janah Shaya[1,2,3], Jean-Charles Ribierre[4], Gabriel Correia[1], Yannick J. Dappe[4], Fabrice Mathevet[5], Loïc Mager[1], Benoît Heinrich[1*], Stéphane Méry[1]*

Experimental

Thermogravimetric analysis (TGA) measurements were carried out with a Q50 apparatus of TA Instruments, at a scanning rate of 5 C min^{-1} and with air as purge gas. Differential scanning calorimetry (DSC) measurements were carried out with a DSCQ1000 apparatus of TA Instruments, at a scanning rate of 5 C min^{-1} on heating and on cooling. Size exclusion chromatography analysis was performed by using 3 columns PLgel mixed D and one column PLgel 100 Å mounted in series (mass separation domain from 200 to 400000 g mol^{-1}) and using a calibration curve from 8 PDMS standards. Optical textures were observed with a Leitz Orthoplan polarizing microscopy equipped with a Mettler FP82 hot stage and a FP80 unit.

The SWAXS patterns were obtained with a transmission Guinier-like geometry. A linear focalized monochromatic Cu Kα1 beam (λ = 1.54056 Å) was obtained using a sealed-tube generator (600 W) equipped with a bent quartz monochromator. The samples were filled in home-made sealed cells of adjustable path. The sample temperature was controlled within ±0.01 °C, and exposure times were varied from 8 h. The patterns were recorded with a curved Inel CPS120 counter gas-filled detector and on image plates scanned by Amersham Typhoon IP with 25 µm resolution. Home-developed software was used to obtain I(2θ) profiles from images and to make empty cell and background subtraction.

GIWAXS measurements were conducted at PLS-II 9A U-SAXS beamline of Pohang Accelerator Laboratory (PAL) in Korea. The X-rays coming from the vacuum undulator (IVU) were monochromated using Si(111) double crystals and focused on the detector using K-B type mirrors. Patterns were recorded with a 2D CCD detector (Rayonix SX165). The sample-to-detector distance was about 225 mm for energy of 11.08 keV (1.119 Å). The samples were spin-coated on silicon wafer from 10 mg/mL solution in chloroform. UV-vis absorption and emission spectra measurements were carried out using a Hitachi U3000 and a Horiba Fluorolog-3 spectrometer, respectively. The PLQY values in films were determined using an integrating sphere system coupled with a photonic multichannel analyzer (Hamamatsu Quantaurus). For each films, the measurements were carried out at four different excitation wavelengths (290, 310, 330 and 350 nm) and the PLQYs reported herein correspond to their average values. It should be reminded that the PLQY is defined as the number of photons emitted as a fraction of the number of photons absorbed and is thus a much more accurate parameter to characterize the emission efficiency than just comparing the maximum emission peak intensity. Time-of-Flight (ToF) measurements were carried out to measure charge carrier mobilities by using the same procedure as previously reported.[1] The samples were prepared using commercial liquid crystal cells (Instec Inc., 8 or 20 µm thick) composed of two ITO-coated glass substrates, that were filled by capillarity under heating at temperatures up to 150°C to obtain liquid materials of low viscosity. For the ToF measurements, the samples were irradiated with a pulsed nitrogen laser at 337 nm. The transient photocurrent signal was searched using a digital storage oscilloscope (Agilent Technologies DSO5034A) by tuning the resistance-capacitance RC time constant of the setup.

The synthesis of the molecules CBP-2Si_3, CBP-4Si_3 and the intermediate steps for CBP-2Si_n were already described in a previous publication.[2] Therefore, only the last step of the preparation of CPB-2Si_n is described below. Monohydrid-terminated oligo(dimethylsiloxane) was purchased from Gelest (MCR-H11). Analysed by SEC (elution in toluene with PDMS standards), this starting compound had the following characteristics : M_n=1240, M_w=1280 and $Đ$=1.20 (see Fig. S1),and it contained an average of 10 dimethylsiloxane units as estimated by ^{1}H-NMR. Karstedt's catalyst represents platinumdivinyl tetramethyldisiloxane complex with 2.0-2.5 % Pt content in xylene. ^{1}H and ^{13}C NMR spectra were recorded on a Bruker Avance 300 and a Bruker 400 Ultrashield^{TM} NMR spectrometers, with an internal lock on the 2H-signal of the solvent. Chemical shifts (δ) are given in ppm. Mass spectra analyses were performed by using a Maldi-TOF (Autoflex II TOF-TOF, Bruker Daltonics, Bremen, Germany) equipped with a nitrogen laser (λ = 337 nm).

4,4'-bis(3-(3-(oligosiloxanyl)propyl)-9H-carbazol-9-yl)biphenyl (CBP-2Si_n). 4,4'-bis(3-allyl-9H-carbazol-9-yl)biphenyl(40.0 mg, 0.071 mmol, 1 eq.) was added to a previously dried reaction tube containing a magnetic bar. Toluene (5.0 mL) and monohydride terminated oligo(dimethylsiloxane) (241 mg, 0.280 mmol, 4 eq.) were added, and the mixture was purged with oxygen for 5 min. Then, the Karstedt's catalyst (50 ppm/SiH) was finally introduced. The mixture was stirred overnight at 90 °C under oxygen atmosphere. The volatiles were evaporated, and the crude product was purified by column chromatography on silica gel with cyclohexane/CH_2Cl_2 as eluent providing the desired product **CBP-2Si_n** as a white pasty wax (137.0 mg, 0.060 mmol, 84%). R_f= 0.50 (Cy/DCM 7:3); ^{1}H NMR (500 MHz, $CDCl_3$, δ): 0.05-0.09 (m, 163H, CH_3), 0.52-0.56 (m, 4H, SiCH_2), 0.65-0.70 (m, 4H, SiCH_2), 0.88 (0.88, 6H, CH_3), 1.30-1.35 (m, 8H, CH_2), 1.74-1.82 (m, 4H, CH_2), 2.85 (t, ^3J = 9.5 Hz, 4H, CH_2), 4.12 (t, ^3J = 7.5 Hz, 4H, ArCH_2), 7.28-7.32 (m, 4H, ArH), 7.41-7.45 (m, 4H, ArH), 7.51 (d, ^3J = 10.0 Hz, 2H, ArH), 7.71 (d, ^3J = 10.5 Hz, 4H, ArH), 7.91 (d, ^3J = 10.5 Hz, 4H, ArH), 7.96 (s, 2H, ArH), 8.15 (d, ^3J = 10.0 Hz, 2H, ArH); ^{13}C NMR (75 MHz, $CDCl_3$, δ): -0.08-0.21 (n-peaks, n-CH_3-Si), 12.8, 16.9, 17.2, 24.4, 25.3, 28.7, 38.8, 108.5, 108.8, 118.8, 118.9, 119.3, 122.5, 122.6, 124.8, 125.8, 126.3, 127.4, 133.6, 136, 5, 138.1, 138.3, 140.0; MALDI (TOF): found 2203.920 ±74.02n.

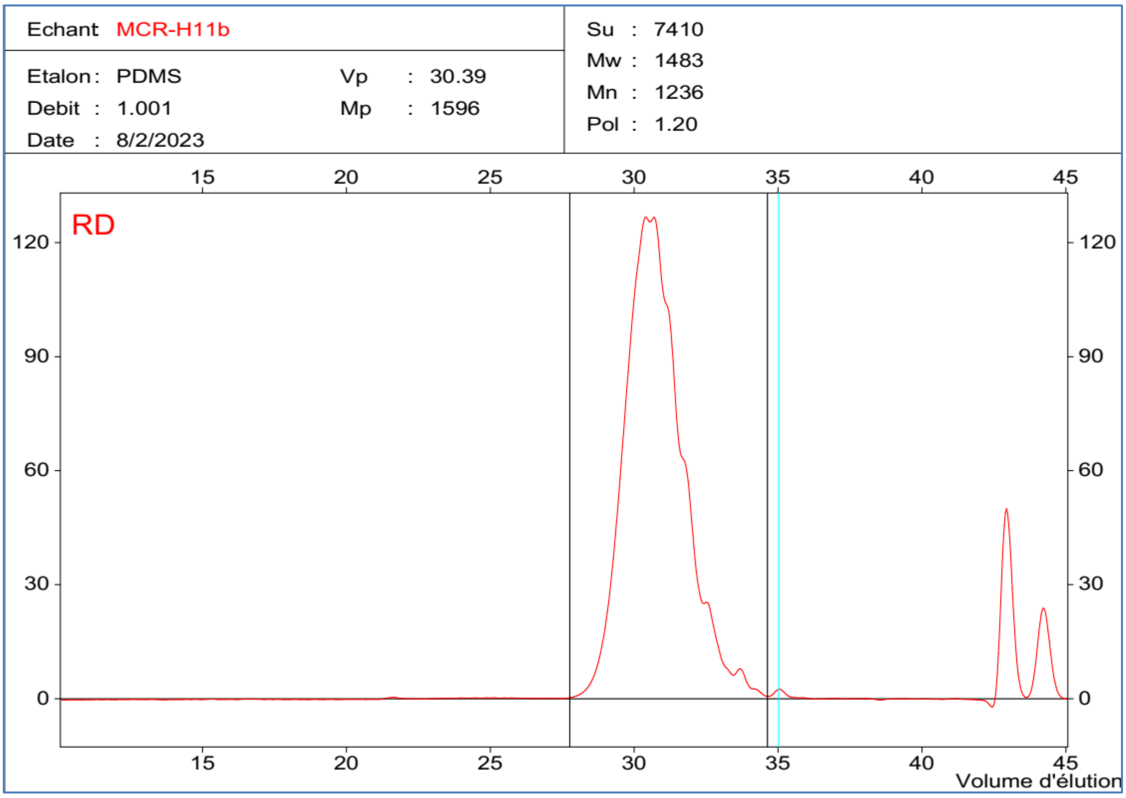

Fig S1. Size exclusion chromatography (SEC) of the starting oligo(dimethylsiloxane) chains (MCR-H11 from Gelest) used in the synthesis of CBP-2Si$_n$. Elution in toluene with PDMS standards ; the peak indicated at 35 mL indicates the octamethylcyclotetrasiloxane reference (D4).

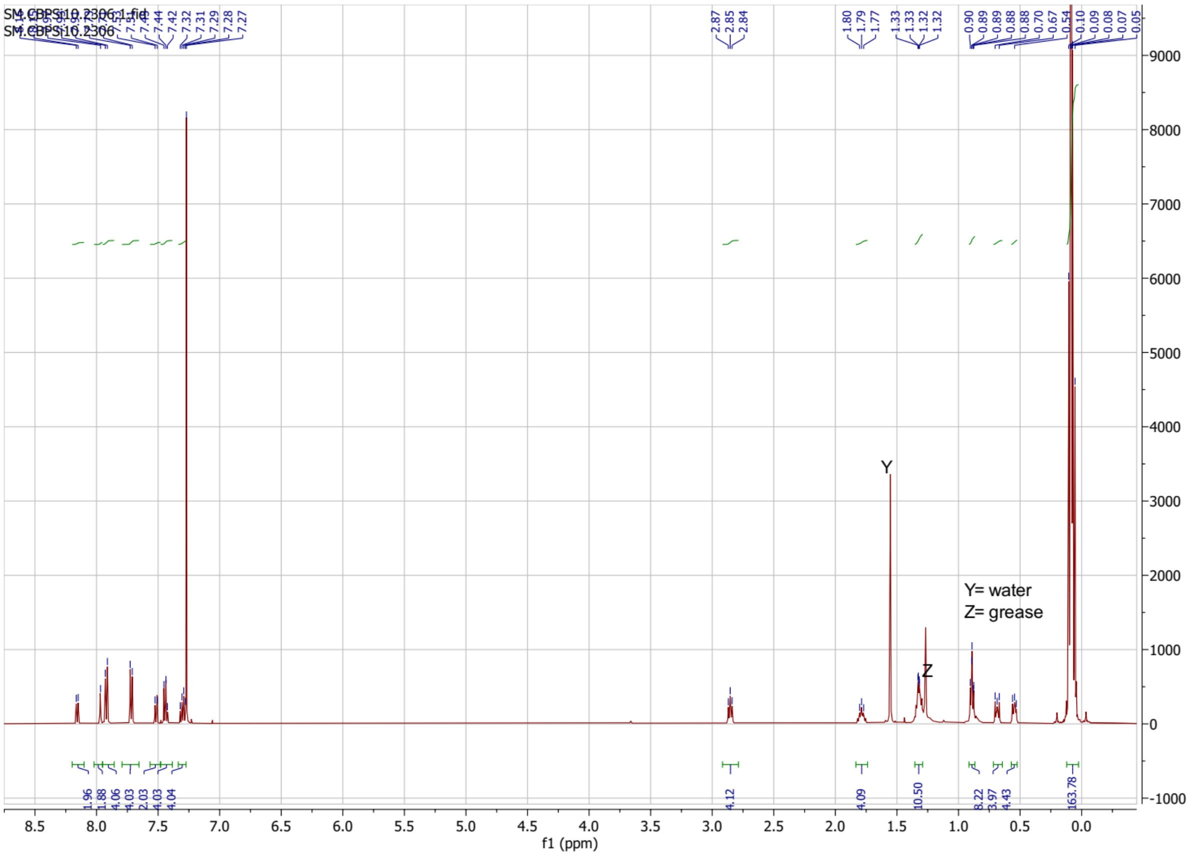

Fig S2. ^{1}H NMR spectrum of CBP-2Si$_n$ recorded in $CDCl_3$.

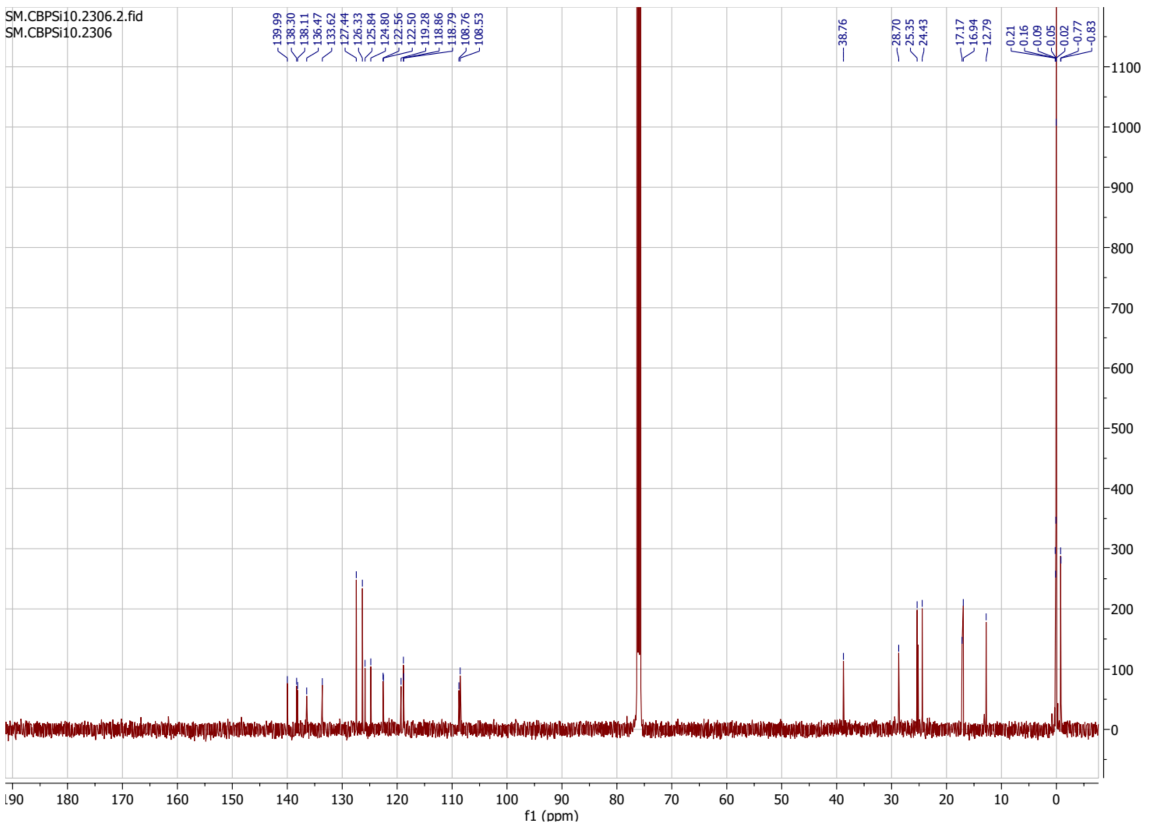

Fig S3. ^{13}C NMR spectrum of CBP-2Si$_n$ recorded in $CDCl_3$.

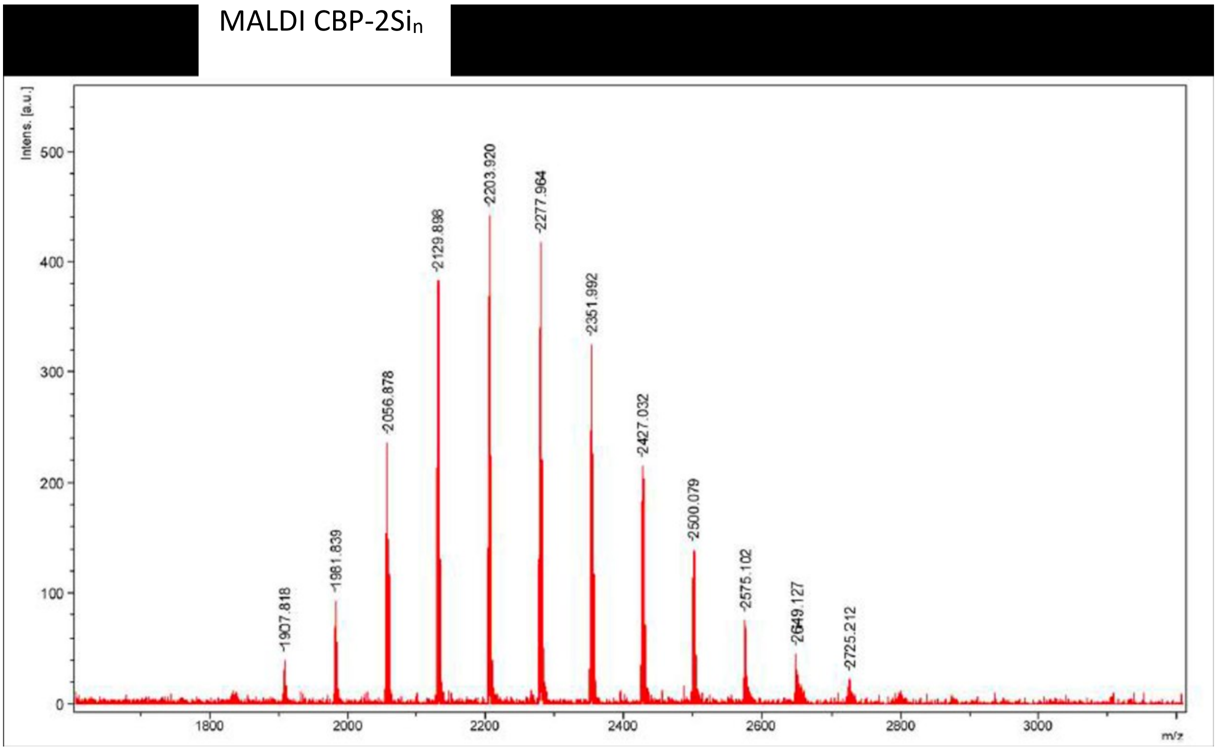

Fig S4. Maldi-ToF MS spectrum of CBP-2Si$_n$.

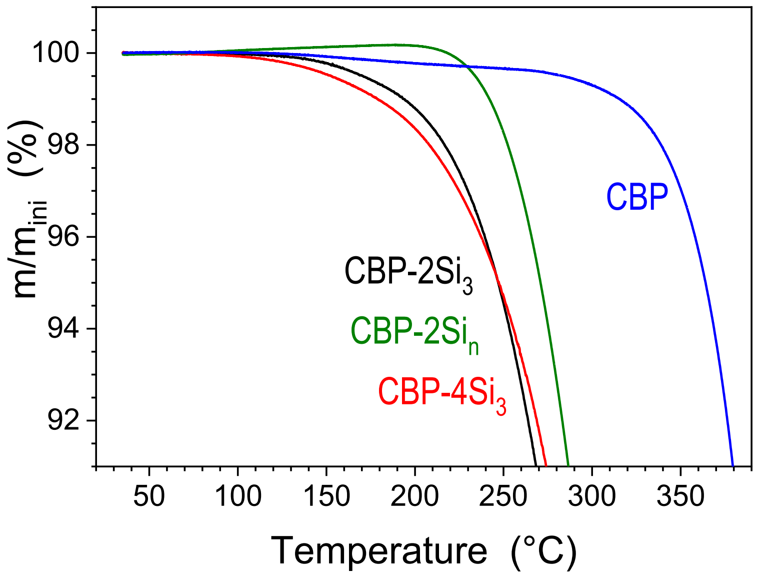

Fig S5. TGA thermograms of CBP and CBP derivatives recorded at 5°C min^{-1}.

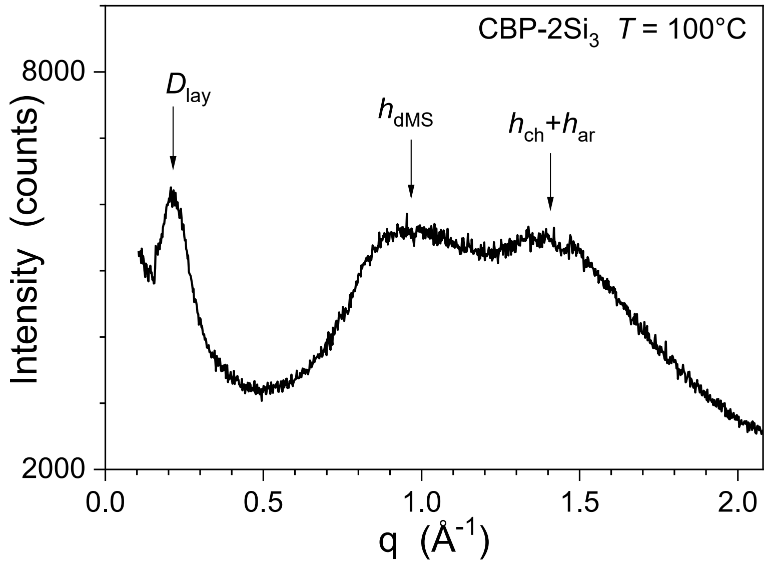

Fig. S6. SWAXS patterns recorded in the liquid phase of CBP-2Si$_3$ (at 100°C). This pattern is similar than the one of CBP-4Si$_3$ recorded in its liquid state at 20°C. Both patterns show distinct scattering signals for siloxane segments (h_{dMS} = 6.5-7 Å), and aliphatic or CBP segments (h_{ch} + h_{ar} = 4.5-5 Å), demonstrating the persistence of nanosegregated strata in the liquid state. The periodicity of the strata alternation leads to a further scattering signal in the low-angle region (D_{lay} = 29 and 27 Å, for CBP-2Si$_3$ and CBP-4Si$_3$, respectively, with similar correlation length $\xi \approx$ 60 Å, determined from Scherrer formula with shape factor K = 0.9.

Table S1. Structural parameters for the CBP layers in the lamellar phases.

Molecule	T(°C)	Phase	a (Ă)	b (Ă)	A (Ă^2)	Z_{2D}	σ_{CBP} (Ă^2)
CBP-2Si$_3$	20	Soft-Cr	20.83	8.74	182.1	2	91.0
CBP-2Si$_n$	10	SmE	21.11	8.87	187.2	2	93.6
	20	SmE	20.72	8.73	180.9	2	90.4

Soft-Cr, SmE: soft crystal (orthorhombic), smectic E-like mesophase; a, b, A, Z_{2D}: lattice parameters, area and number of mesogens per lattice or sub-lattice; $\sigma_{CBP} = A/Z_{2D}$: cross-sectional area of a side-attached CBP mesogen.

Table S2. Siloxane layer configurations in the nanosegregated phases.

Molecule	T(°C)	phase	V_{mol} (Ă^3)	ρ (g/cm^3)	d_{lam} (Ă)	A_{mol} (Ă^2)	a_{dMS} (Ă^2)	τ_{dMS}
CBP-2Si$_3$	20°C	Soft-Cr	1152	1.080	17.25	90.0	90.0	0.00
	100°C	Liq	1645	1.019	28.8	57.1	57.1	0.73
CBP-4Si$_3$	20°C	Liq	2454	1.039	27.3	89.9	45.0	1.00
CBP-2Si$_n$	10°C	SmE	3569	1.026	48.10	74.2	74.2	0.35
	20°C	SmE	3611	1.015	46.10	78.3	78.3	0.26
	20°C	Liq	3611	1.015	41.71	86.6	86.6	0.08

Soft-Cr (soft crystal), Liq (isotropic liquid phase), SmE (smectic E-like mesophase).

V_{mol}, ρ: molecular volume and density, calculated from database of reference measurements.

d_{lam}, lamellar periodicity in the soft crystal: $d_{lam} = d_{002}$; SmE phase: $d_{lam} = d_{001}$; strata periodicity in the isotropic liquid phase: $d_{lam} = D_{lay}$.

$A_{mol} = V_{mol}/d_{lam}$: molecular area, i.e. the average area covered by one molecule in each sublayer of the lamellar sequence.

$a_{dMS} = A_{mol}$: area per CBP unit (1 BCP segment per molecule and 1 CBP monolayer par lamella).

$a_{dMS} = A_{mol} \times n_{LdMS}/n_{dMS}$: area per siloxane segment, n_{dMS} being the number of siloxane chains per molecule ($n_{dMS} = 2$ for CBP-2Si$_3$ and CBP-10Si$_3$; $n_{dMS} = 4$ for CBP-4Si$_3$) and $n_{LdMS} = 2$, the number of sublayers in which these chains are involved.

σ_{dMS}: cross-sectional area of a siloxane chains, determined from the ratio of the experimentally measured repeat unit volume of PDMS and the repeat unit length of a completely stretched chain ≈ 3.10 Å.[3] Therefore, the lower limit of the cross-sectional area of a siloxane chain is: $\sigma_{dMS} = 41\cdot(1 + 9.2 \times 10^{-4}\,(T - 20))$ Å^2. However, contrarily to alkyl chains crystallizing in all-trans conformation, siloxane chains are never completely stretched, even in single crystal structures of oligodimethylsiloxane containing molecules. In practice, it is observed that successive siloxane units adopt rotated conformations with respect to each other, reducing somewhat the apparent repeat unit length along the average chain axis and expanding σ_{dMS} in proportion.

$\tau_{dMS} = 2 - a_{dMS}/\sigma_{dMS}$: calculated bilayer ratio parameter varying between 0 for a total intercalation of adjacent molecular layers ($a_{dMS} \approx 2\sigma_{dMS}$) and 1 for a superposition into a perfect bilayer arrangement ($a_{dMS} \approx \sigma_{dMS}$). For the flat layers of CBP-2Si$_3$ soft-crystal, the configuration is obviously intercalated,

implying $\tau_{dMS} = 0$ and thus $\sigma_{dMS} = 45$ Å^2. This value is slightly higher than the low-limit value of 41 Å^2 and corresponds to an apparent repeat unit length of 2.8 Å, in accordance with the slightly under-stretched chain conformations observed in single crystal structures. This effect was considered in the calculation of τ_{dMS}, by using the following expression for the apparent cross-sectional area of siloxane chains: $\sigma_{dMS} \approx 45 \cdot (1 + 9.2 \times 10^{-4}\,(T - 20))$ Å^2.

The variation of τ_{dMS} evidences the evolution of the siloxane layer configuration. τ_{dMS} raises to value between 0 and 1 in the liquid phase of CBP-2Si$_3$, which follows the partial disentanglement of the siloxane chains, and equals 1 for CBP-4Si$_3$ since siloxane chains adopt a bilayer configuration. For CBP-2Si$_n$, the value in the liquid is close to 0 indicating an intercalated monolayer but raise above this value in the smectic phase. As a matter of fact, these long chains are polydisperse, which promotes a partial disentanglement of the chains towards a partial bilayer configuration. A schematic representation of this evolution is given in figure S7.

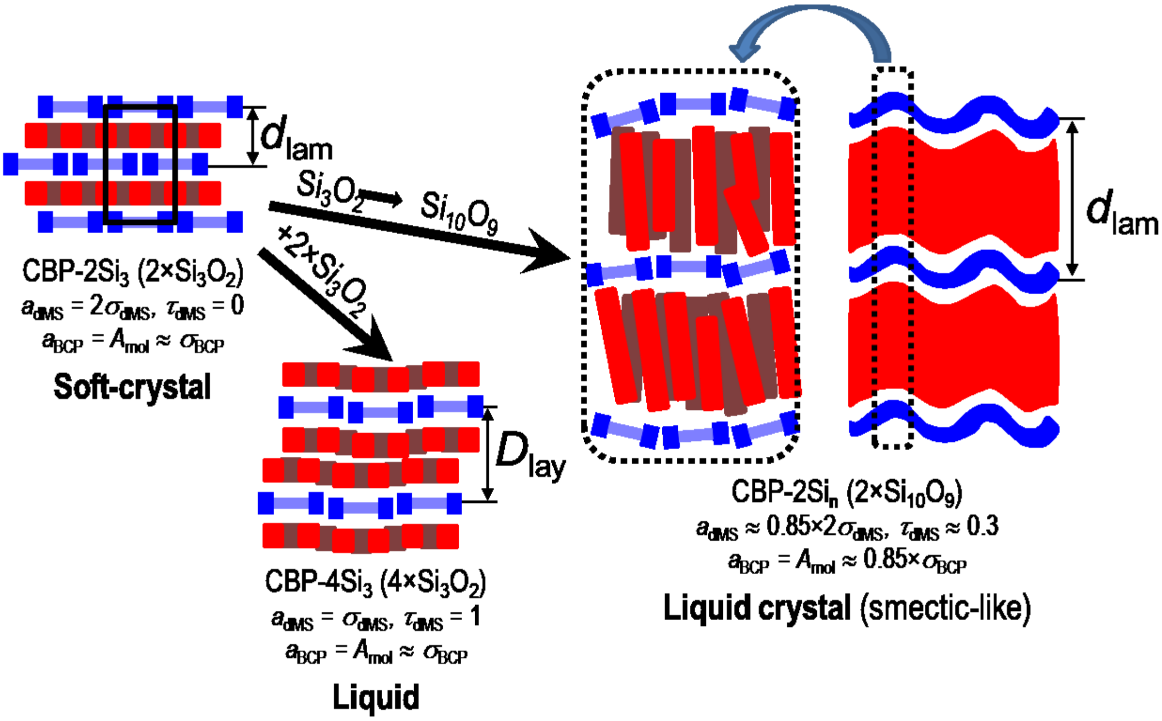

Fig. S7. Illustration of the evolution of the self-organized structures of the CBP derivatives as a function of the siloxane chains content, including molecular parameters issued from Table S5.

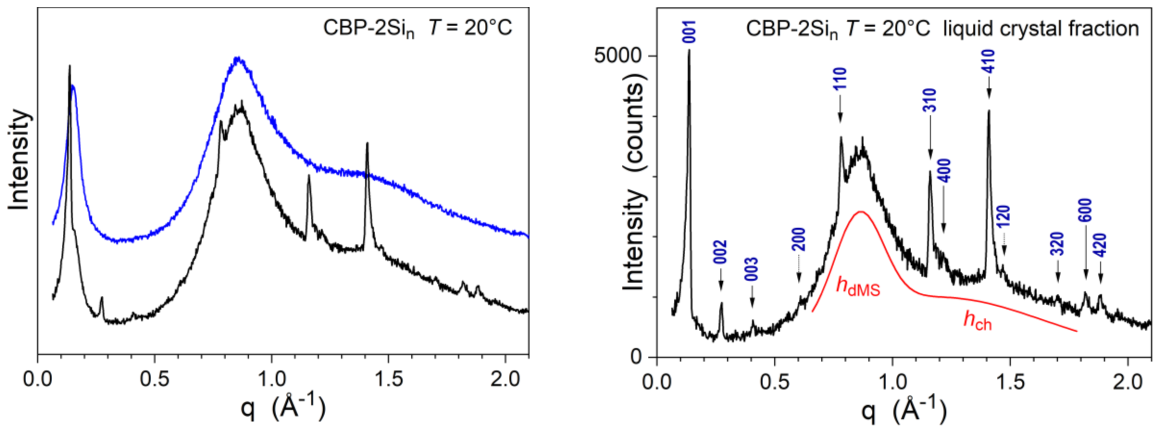

Fig. S8. Left: SWAXS patterns of the CBP-2Si$_3$ in the pristine state at 20°C (black curve) and in the supercooled liquid phase at 20°C (blue curve). Right: SWAXS pattern of CBP-2Si$_3$ in the mesophase at 20°C, after subtraction of the 43% liquid phase amount present in the pristine state.

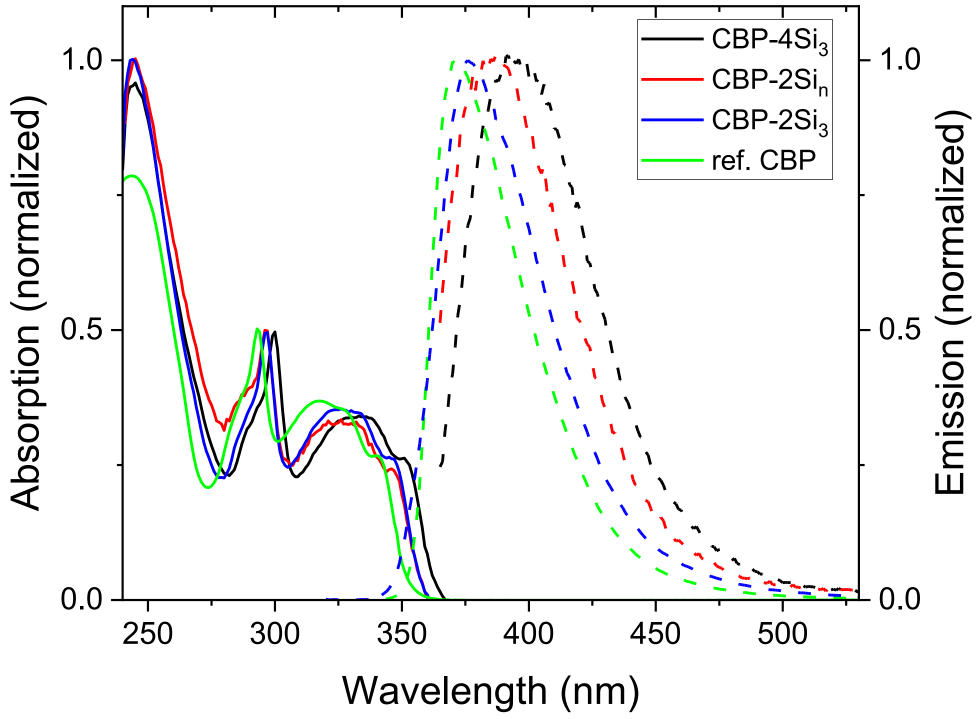

Fig. S9. Absorption and emission (under excitation at 310 nm) spectra of the CBP derivatives in solution (10^{-5} M in dichloromethane).

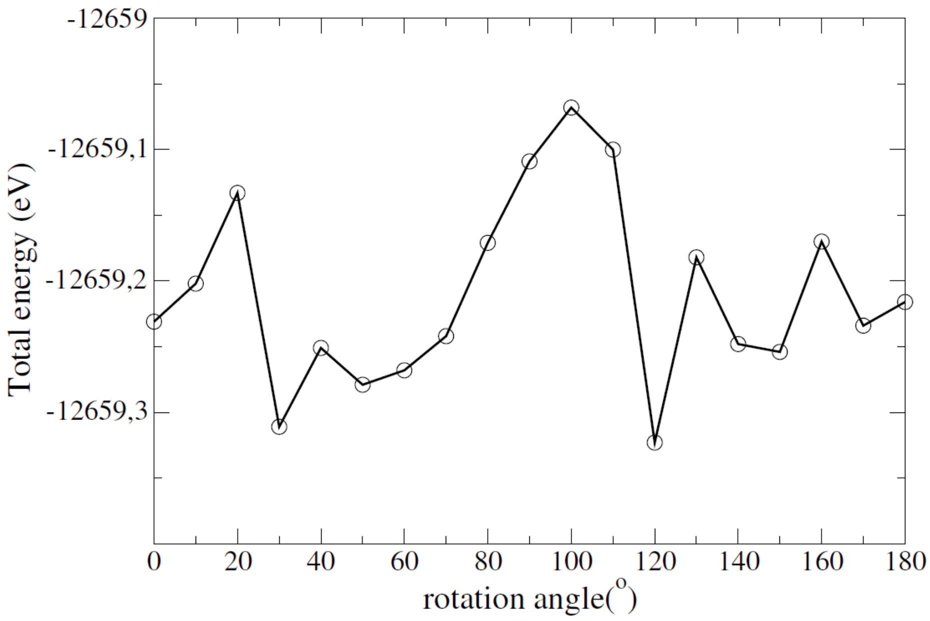

Fig. S10. Potential energy landscape of the CBP-2Si_3 molecule calculated at the DFT level by varying the torsion angle between the two N-phenylcarbazole units.

[1] J.C. Ribierre; L. Bao, M. Inoue, P.O. Schwartz, J.-H. Kim, K. Yoshida, A.S.D. Sandanayaka, H. Nakanotani, L. Mager, S. Méry, C. Adachi. *Chem. Commun*. **2016**, *52*, 3103-3106.

[2] J. Shaya, G. Correia, B. Heinrich, J.-C. Ribierre, K. Polychronopoulou, L. Mager, S. Méry, *Molecules* **2022**, *27*, 89.

[3] N. Kamatham, O. A. Ibraikulov, P. Durand, J. Wang, O. Boyron, B. Heinrich, T. Heiser, P. Lévêque, N. Leclerc, S. Méry, *Adv. Funct. Mater*. **2020**, 2007734.
